# Supplementary material for: Chiral gold nanoparticles enantioselectively rescue memory deficits in a mouse model of Alzheimer’s disease
Source: Nat Commun. 2020 Sep 22;11:4790. doi: 10.1038/s41467-020-18525-2 (PMC7509831; doi:10.1038/s41467-020-18525-2)
Supplement: Supplementary file 1 — Supplementary Information [file 41467_2020_18525_MOESM1_ESM.pdf]

Supplementary Information

Chiral Gold Nanoparticles Enantioselectively Rescue Memory Deficits in a Mouse Model of Alzheimer's Disease

Hou et al.

Supplementary Method

Monte Carlo simulation

In Monte Carlo simulation, a linear chain is adopted to represent amyloid peptide molecule. Each chain includes 10 coarse-grained (CG) beads and the bead size σ is set as 0.23 nm. The bond length between two adjacent beads in the same chain is 0.32 nm, so the length of the peptide chain is 3.2 nm if it is fully stretched, which is almost the same with the width of one Aβ42 molecule[1]. The non-bonded interaction between peptide beads can be represented via a repulsive potential[2],

$$U_{\mathrm{rep}} = 4\varepsilon_{\mathrm{rep}} \left(\frac{\sigma_{\mathrm{rep}}}{r}\right)^{12} \tag{1}$$

Where $\varepsilon_{\mathrm{rep}}$ is the interaction parameter and set to 1.3 kJ mol^{-1} and σ_{rep} is the bead size, The rigidity of the chain is represented by a three-body angle potential[2,3],

$$U_{\mathrm{rigid}} = g_i(1 + \cos\theta)^2 \tag{2}$$

where $\cos\theta = \left((\vec{\mathbf{r}}_{i+1} - \vec{\mathbf{r}}_i) \cdot (\vec{\mathbf{r}}_{i-1} - \vec{\mathbf{r}}_i)\right)/{r_0}^2$, g_i is the rigidity constant and set to 258 kJ mol^{-1}, which means a stiff backbone, and r_0 is the bond length.

Driven by hydrogen bond interaction between peptide chains, the peptide molecules can self-assemble into fibrils. In 2008, Velichko and co-workers developed a very simple and efficient approach to mimic hydrogen bonding[3], and it was implemented to Monte Carlo method in 2012[2]. Detailly, the saturation of forming hydrogen bond for a CG bead is set to 2, since there are one donor N-H and one acceptor C=O in each amino acid. The hydrogen bond is allowed to form or break during the simulation. The activation energy for forming the first hydrogen bond of CG bead is defined as[4],

$$U_a = \varepsilon_\beta \cos(\vartheta_{ij}) \tag{3}$$

Where ε_β is the hydrogen bond energy, which determines the probability of forming and breaking hydrogen bonds and is set to 7.73 kJ mol^{-1}. The υ_{ij} is the angle between the bonding CG beads in the two neighbor chains, $\cos(\vartheta_{ij}) = (\Delta\vec{\mathbf{r}}_i \cdot \Delta\vec{\mathbf{r}}_j)/(\Delta r_i \Delta r_j)$, in which $\Delta\vec{\mathbf{r}}_i = \vec{\mathbf{r}}_{i+1} - \vec{\mathbf{r}}_{i-1}$, the bead i and j belong to different chains, thus the two peptide chains tend to be parallel with each other according to the Supplementary equation 3 (Supplementary Fig. 1a). If the CG bead (bead i) can form the second hydrogen bond, the CG bead k from another chain will be placed along the direction of the CG beads i and j. We add an additional angle φ_i for the second hydrogen bond, as the β-sheet structure requires a flat geometry, which is also shown in Supplementary Fig. 1a. The activation

energy for the second hydrogen bond is,

$$U_a = \varepsilon_\beta \cos(\vartheta_{ij}) \cos(\varphi_i) \tag{4}$$

where φ_i is defined as $\cos(\varphi_i) = (\Delta\vec{\mathbf{r}}_{ki} \cdot \Delta\vec{\mathbf{r}}_{ij})/(\Delta r_{ki}\Delta r_{ij})$. The probabilities for forming and breaking hydrogen bonds, p_{form} and p_{break} respectively, are determined by Metropolis criterion displayed below,

$$p_{\text{form}} = \frac{g(U_{a,ij})}{1+g(U_{a,ij})} \tag{5}$$

$$p_{\text{break}} = \frac{1}{1+g(U_{a,ij})} \tag{6}$$

where $g(U_{a,ij}) = \exp(-U_{a,ij}/k_{\text{B}}T)$, $U_{a,ij}$ is the activation energy of hydrogen bonds. If the distance between the two CG beads is smaller than 0.35 nm, a random number R between 0 and 1 could be generated; if R is smaller than p_{form} or p_{break}, the hydrogen bond can form or break.

The Au NPs are represented by a hollow sphere model, which is made up of many small CG beads (Supplementary Fig. 1b). The number of beads in one NP is determined by the size of NPs and is adjusted to keep the number per unit area same. The number of NPs distributed in the simulation boxes depends on the NP size with the same total surface area. In our simulation, six different sized NPs have been selected to interact with peptide chains and the detailed information for NPs is shown in Supplementary Table 1. The pure peptide solution system without NPs is also built as comparison. The size of the small beads σ in the NP is same as that of peptide beads, 0.23 nm, and the interaction between NP beads and peptide beads is described via Lennard-Jones (LJ) potential,

$$U = 4\varepsilon_{\text{np}}\left(\left(\frac{\sigma}{r}\right)^{12} - \left(\frac{\sigma}{r}\right)^{6}\right) \tag{7}$$

where ε_{np} is the interaction parameter between peptide beads and NP beads and is set as 3.87 kJ mol^{-1}, so the peptide chains could be adsorbed on the NP surfaces driven by the attractive LJ potential. In addition, the hard-sphere potential between the centers of spherical NPs and peptide beads is adopted, in order to prevent the peptide chains from penetrating the surface and moving into the NP. The hard-sphere potential is described as,

$$U_{\text{HS}} = \begin{cases} +\infty, & r \le d_{\text{np}} \\ 0, & r > d_{\text{np}} \end{cases} \tag{8}$$

where d_{np} is the diameter of NP. The implicit solvent model is used in the system, which provides a good solvent condition for the peptide chains.

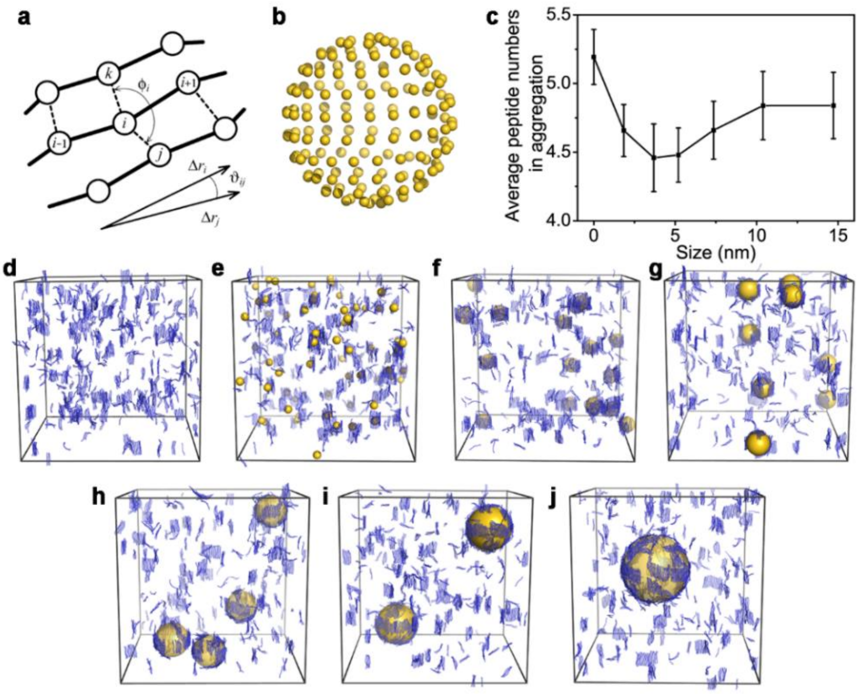

Supplementary Figure 1. Monte Carlo simulation about the effect of NP size on peptide aggregation. a, Schematic diagram of hydrogen bonds in the coarse-grained (CG) model of polypeptides. Three beads *i*, *j*, *k* form two hydrogen bonds, which are shown in dashed lines. The υ_{ij} is the angle between the bonding CG beads in the two neighbor chains. An additional angle φ_i represents the second hydrogen bond, as the β-sheet structure requires a flat geometry. Adapted with permission from Supplementary Reference 2. Copyright 2012 American Chemical Society. **b**, Representative spherical NP with a diameter of 3.8 nm. It includes 160 small beads and the beads are shown as gold color. **c**, Plot of average numbers of CG model of polypeptides in the formed aggregate against spherical NPs with different sizes. Mean ± s.d. values are 5.19 ± 0.20, 4.66 ± 0.19, 4.46 ± 0.25, 4.48 ± 0.20, 4.66 ± 0.21, 4.84 ± 0.25, 4.84 ± 0.24 for peptide solution alone and in the presence of NPs with a diameter of 1.84 nm, 3.68 nm, 5.20 nm, 7.36 nm, 10.40 nm and 14.72 nm. (n = 10 independent samples). **d-j**, Simulation results of peptide solution alone (**d**) and in the presence of NPs with a diameter of 1.84 nm (**e**), 3.68 nm (**f**), 5.20 nm (**g**), 7.36 nm (**h**), 10.40 nm (**i**) and 14.72 nm (**j**). Source data are provided as a Source Data file.

The presence of Au NPs considerably decreases the average numbers of CG model of polypeptides in the formed aggregate in comparison with peptide solution alone, and Au NPs with a size of 3~4 nm has the best performance (Supplementary Fig. 1c,f).

Supplementary Table 1. Parameter of different sized NPs used in simulation.

Diameter (nm)	Beads in one NP	Number of NPs
1.84	40	64
3.68	160	16
5.20	320	8
7.36	640	4
10.40	1280	2
14.72	2560	1

The number of beads in one NP is determined by the size of NPs and is adjusted to keep the number per unit area the same. The number of NPs distributed in the simulation boxes depends on the NP size with the same total surface area (Supplementary Fig. 1).

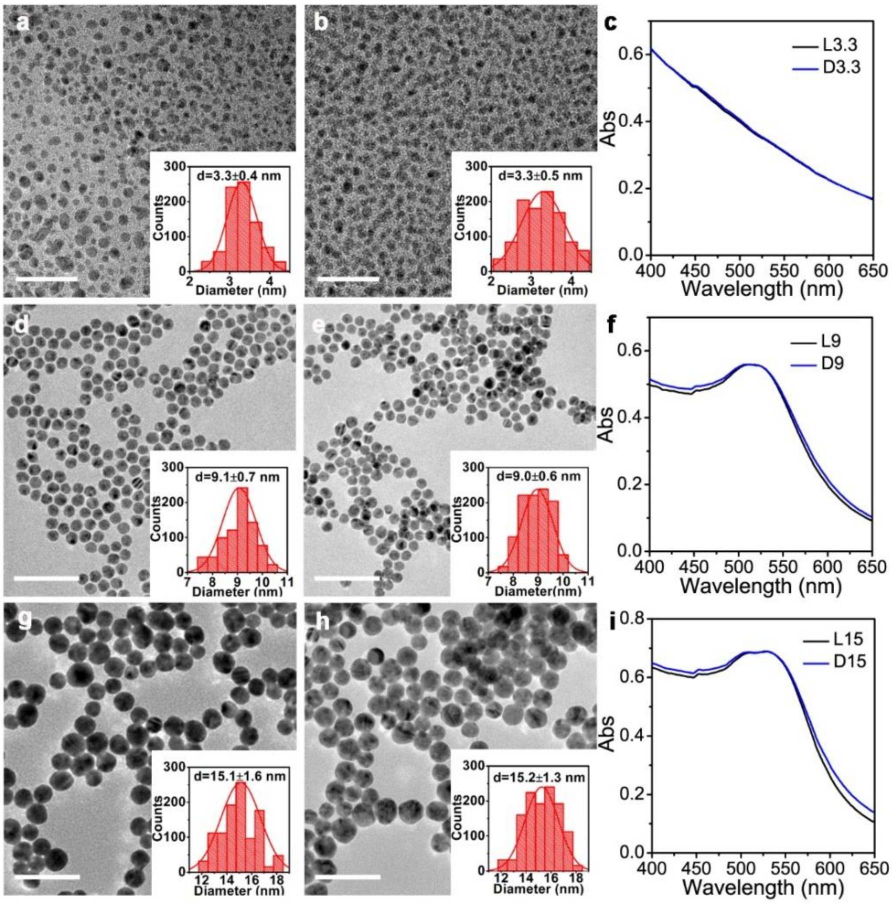

Supplementary Figure 2. Morphology and spectral characterization of as-synthesized L- and D-GSH stabilized Au NPs. **a**,**b**, TEM images of L3.3 (**a**) and D3.3 (**b**). Scale bars, 20 nm. **c**, UV-vis absorption spectra of L3.3 and D3.3. **d**,**e**, TEM images of 9 nm L-GSH capped Au nanoparticles (denoted as L9) (**d**) and 9 nm D-GSH capped Au nanoparticles (D9) (**e**). Scale bars, 50 nm. **f**, UV-vis absorption spectra of L9 and D9. **g**,**h**, TEM images of 15 nm L-GSH capped Au nanoparticles (denoted as L15) (**g**) and 15 nm D-GSH capped Au nanoparticles (denoted as D15) (**h**). Scale bars, 50 nm. **i**, UV-vis absorption spectra of L15 and D15. The inset shows size distribution histograms (n = 1000 nanoparticles). Source data are provided as a Source Data file.

The TEM images reveal that as-synthesized chiral L- or D-GSH coated Au NPs are highly uniform with narrow size distribution (Supplementary Fig. 2a,b,d,e,g,h). In addition, the UV-vis absorption spectra of L3.3, L9 and L15 overlap with those of D3.3, D9 and D15 (Supplementary Fig. 2c,f,i). L9, D9, L15 and D15 all possess strong adsorption peaks at 525 nm. As comparison, the typical surface plasmon resonance bands of Au NPs are not observed in L3.3 and D3.3 (Supplementary Fig. 2c), which are caused by their small size.

Supplementary Table 2. Physicochemical characteristics of as-prepared 3.3 nm, 9 nm and 15 nm L- and D-GSH stabilized Au NPs obtained by dynamic light scattering measurement.

Samples	Hydrodynamic diameter (nm)	Zeta potential (mV)
L3.3	7.2 ± 2.3	-43.2 ± 10.3
D3.3	7.3 ± 2.1	-42.0 ± 6.1
L9	16.7 ± 6.5	-45.5 ± 10.6
D9	16.1 ± 6.5	-45.2 ± 7.6
L15	24.4 ± 7.7	-37.3 ± 6.0
D15	24.3 ± 7.8	-37.0 ± 5.6

As-synthesized L3.3, L9 and L15 have almost the same hydrodynamic diameters and zeta potential values with D3.3, D9 and D15, respectively.

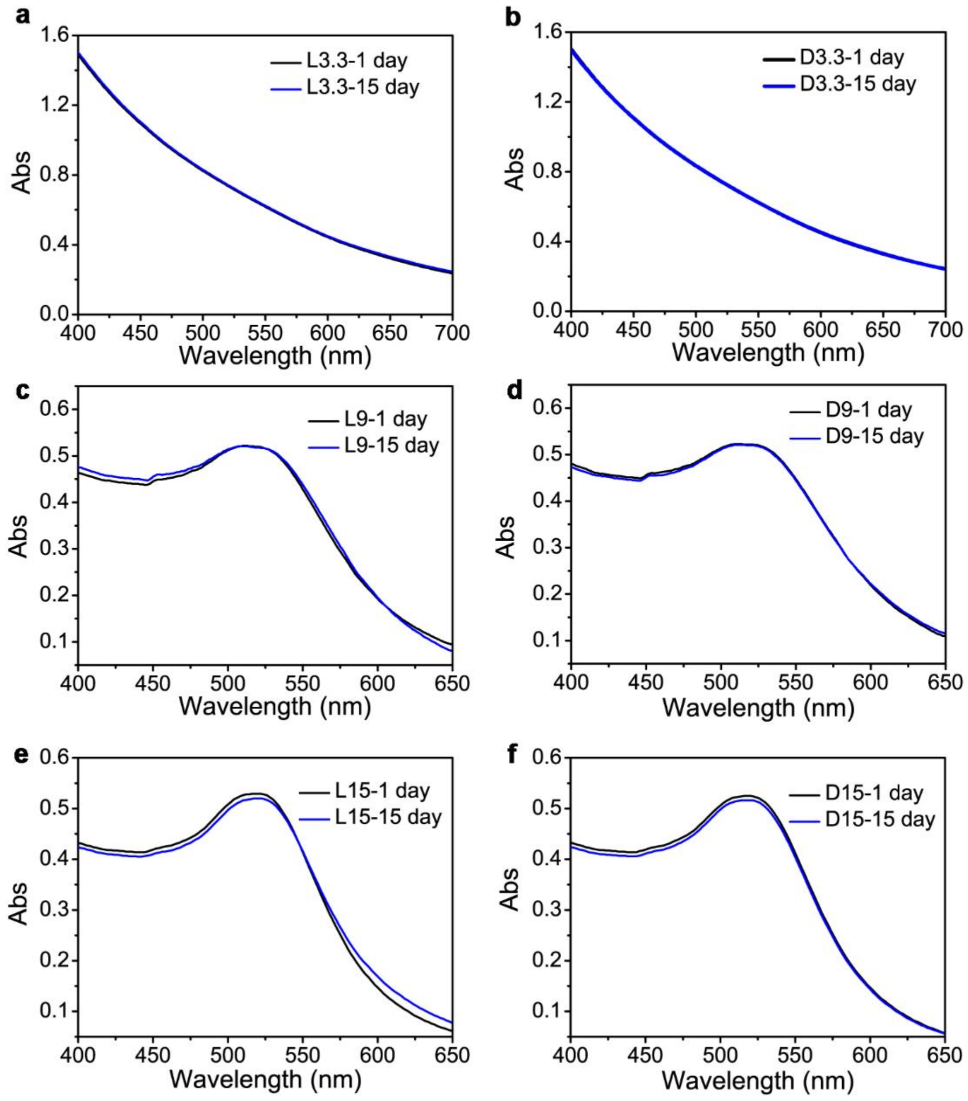

Supplementary Figure 3. Stability of chiral L- and D-GSH coated Au NPs in PBS. **a-f**, UV-vis absorption spectra of L3.3 (**a**), D3.3 (**b**), L9 (**c**), D9 (**d**), L15 (**e**), D15 (**f**) after dispersed in PBS for 1 day and 15 days, respectively. Source data are provided as a Source Data file.

As evidenced in Supplementary Fig. 3, the obtained L3.3, D3.3, L9, D9, L15, and D15 exhibit excellent stability in PBS and no obvious aggregation is observed even after 15 day storage, which will benefit their biological applications.

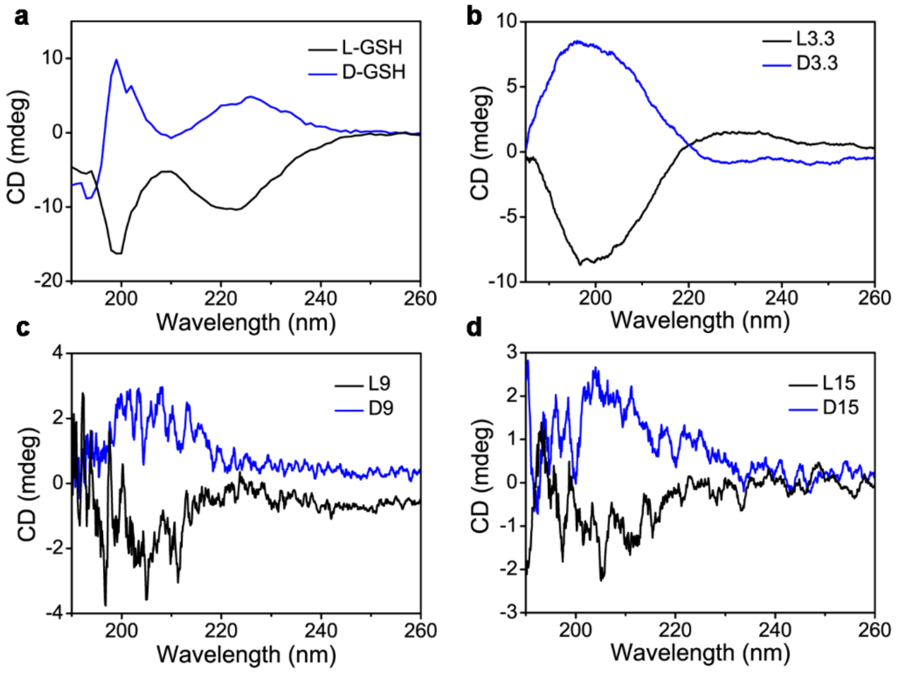

Supplementary Figure 4. Optical activity of chiral L- and D-GSH coated Au NPs. a-d, CD spectra of chiral L-GSH and D-GSH molecules (**a**), L3.3 and D3.3 (**b**), L9 and D9 (**c**), L15 and D15 (**d**). Source data are provided as a Source Data file.

The negative peaks at ~200 nm appear in the CD spectra of L3.3, L9, and L15, whereas L3.3, L9, and L15 show positive CD signals, indicating the chiral nature of these Au NPs Considering that as-synthesized chiral Au NPs have almost the same size, light absorption (Supplementary Fig. 2), hydrodynamic diameters and surface charge (Supplementary Table 2), it is easily concluded that the surface chirality is the only difference between L- and D-GSH coated Au NPs.

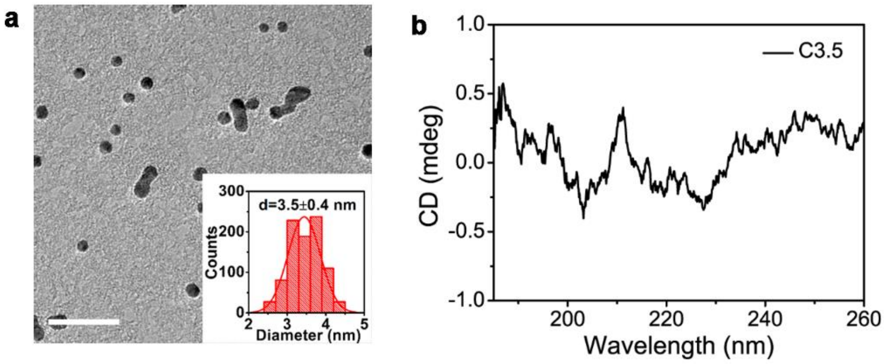

Supplementary Figure 5. Characterization of as-synthesized C3.5. a, TEM image of C3.5. Inset is the histogram of NP size distribution (n = 1000 nanoparticles). Scale bar, 20 nm. **b**, CD spectrum of C3.5. Source data are provided as a Source Data file.

As demonstrated in Supplementary Fig. 5, 3.5 nm citrate stabilized Au NPs are successfully synthesized and they don't show optical activity in the CD spectrum.

Supplementary Table 3. Extinction coefficient ε and concentration of 3.3 nm, 9 nm and 15 nm L- and D-GSH coated Au NPs incubating with Aβ42 in vitro.

Sample	Extinction coefficient ε[a] ($M^{-1}\cdot cm^{-1}$)	Concentration[b] (nM)
L3.3/D3.3	3.2×10^{6}	110
L9/D9	5.0×10^{7}	14.8
L15/D15	2.0×10^{8}	5.4

[a]The extinction coefficient ε of L- and D-GSH coated Au NPs with different sizes is obtained according to the equation[5], $\ln\varepsilon = k\ln D + a$, where $k = 2.75$, $a = 11.7$ and D is the diameter of NPs. [b]Since Au NP surface area might affect the adsorption of Aβ42, the concentration of L9/D9 and L15/D15 is adjusted to have the same surface areas with L3.3 and D3.3 in the experiment.

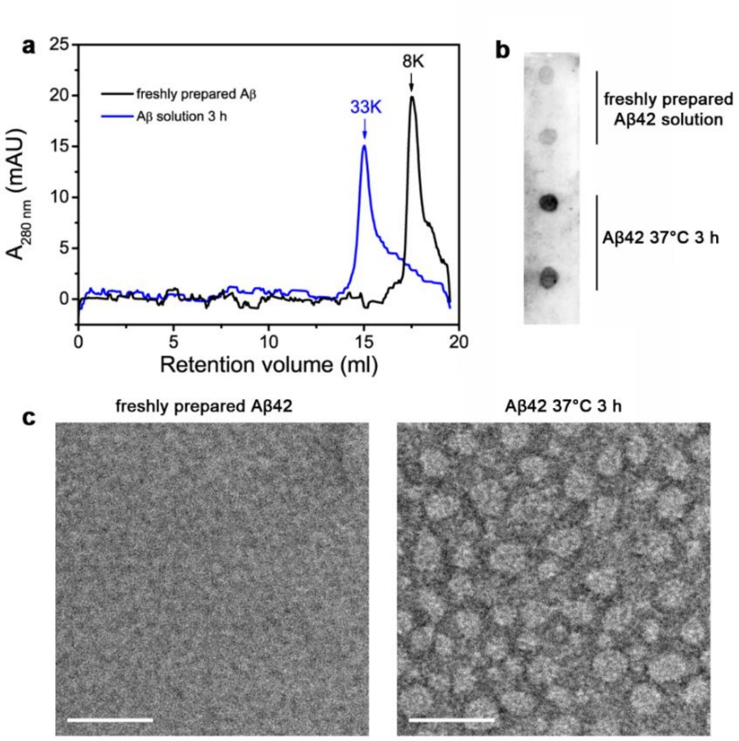

Supplementary Figure 6. Validation of Aβ42 preparation. a-c, Size exclusion chromatography (**a**), dot-blot assay (**b**) and TEM images (**c**) of freshly prepared Aβ42 solution and Aβ42 solution incubated at 20 μM for 3 h. Scale bars, 50 nm. The dot-blot assay and TEM characterization were repeated three times with similar results. Source data are provided as a Source Data file.

As seen in Supplementary Fig. 6a, the molecular weight of Aβ42 form in the freshly prepared solution is about 8 kDa, which corresponds to that of Aβ42 monomer reported before[6]. After the Aβ42 solution incubated at 37°C for 3 h, the molecular weight of Aβ42 forms increases to 33 kDa, indicating the formation of Aβ42 oligomers[6] (Supplementary Fig. 6a). To better identify the nature of Aβ42 forms in these two conditions, dot-blot analysis with A11 antibodies have been conducted. A11 is an antibody reported to selectively recognize soluble amyloid oligomers and prefibrillar aggregates[7]. As seen in Supplementary Fig. 6b, the freshly prepared Aβ42 solution produces a very little signal. In contrast, the Aβ42 sample generated after three hours of incubation at 37°C shows strong positive signals, implying that the Aβ42 solution prepared by our protocol remains monomer instead of oligomers. This conclusion can be further verified by TEM images (Supplementary Fig. 6c). No large aggregates are observed in freshly prepared Aβ42 solution. Meanwhile, Aβ42 incubated at 37°C for 3 h generates sphere-shaped aggregates.

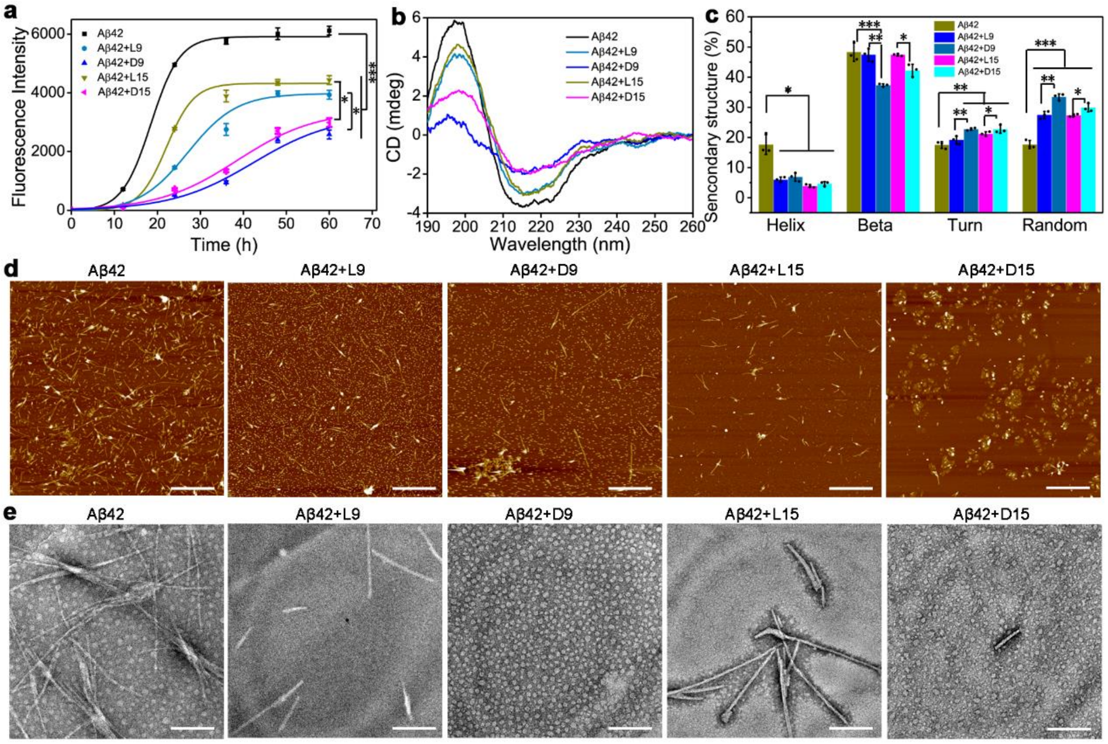

Supplementary Figure 7. Effect of L9, D9, L15, and D15 on Aβ42 fibrillization in vitro. a, ThT fluorescence assay of Aβ42 in the absence and presence of L9, D9, L15 and D15. Aβ42 + L9, Aβ42 + D9, Aβ42 + L15 and Aβ42 + D15 represents Aβ42 incubated with L9, D9, L15 or D15, respectively. The fibrillation kinetics is fitted with a sigmoidal function. Mean ± s.d. values are 14.3 ± 7.8, 718.7 ± 30.7, 4960.0 ± 50.1, 5764.3 ± 100.3, 6016.7 ± 199.9, 6125.0 ± 150.0 at 0, 12, 24, 36, 48, 60 h for Aβ42. Mean ± s.d. values are 8.3 ± 4.0, 145.7 ± 47.0, 1460.3 ± 40.0, 2756.3 ± 199.8, 3974.0 ± 99.8, 3938.3 ± 151.2 at 0, 12, 24, 36, 48, 60 h for Aβ42+L9. Mean ± s.d. values are 20.3 ± 18.9, 159.3 ± 49.2, 2781.3 ± 40.5, 3894.0 ± 199.5, 4362.0 ± 99.7, 4441.7 ± 149.6 at 0, 12, 24, 36, 48, 60 h for Aβ42+D9. Mean ± s.d. values are 10.0 ± 4.6, 125.3 ± 30.2, 527.3 ± 40.1, 958.3 ± 49.7, 2403.3 ± 99.2, 2585.7 ± 150.0 at 0, 12, 24, 36, 48, 60 h for Aβ42+L15. Mean ± s.d. values are 15.0 ± 11.5, 128.0 ± 30.1, 715.0 ± 39.8, 1346.0 ± 50.3, 2705.0 ± 100.1, 2991.0 ± 150.0 at 0, 12, 24, 36, 48, 60 h for Aβ42+D15. **b**, CD spectra of Aβ42 (40 μM) in the absence and presence of L9, D9, L15 or D15 after co-incubation for 48 h. **c**, Analysis of protein secondary structure. Mean ± s.d. values of helix structure are 17.6 ± 3.3%, 6.0 ± 0.9%, 6.9 ± 1.4%, 3.9 ± 0.5%, 4.6 ± 0.8% for Aβ42, Aβ42+L9, Aβ42+D9, Aβ42+L15, Aβ42+D15. Mean ± s.d. values of beta structure are 48.3 ± 3.0%, 47.4 ± 2.2%, 37.3 ± 0.5%, 47.4 ± 0.4%, 42.2 ± 2.1% for Aβ42, Aβ42+L9, Aβ42+D9, Aβ42+L15, Aβ42+D15. Mean ± s.d. values of turn structure are 17.5 ± 1.1%, 19.1 ± 1.4%, 22.7 ± 0.4%, 21.2 ± 0.7%, 22.7 ± 1.4% for Aβ42, Aβ42+L9, Aβ42+D9, Aβ42+L15, Aβ42+D15. Mean ± s.d. values of

random structure are 17.6 ± 1.4%, 27.4 ± 1.2%, 33.4 ± 1.1%, 27.3 ± 0.4%, 29.9 ± 1.4% for Aβ42, Aβ42+L9, Aβ42+D9, Aβ42+L15, Aβ42+D15. **d**, AFM images of Aβ42 (40 μM) in the absence and presence of L9, D9, L15 or D15 after co-incubation for 48 h. Scale bars, 1 μm. **e**, TEM images of Aβ42 (40 μM) in the absence and presence of L9, D9, L15 or D15 after co-incubation for 48 h. Scale bars, 200 nm. Error bars indicate the standard deviation (s.d.) (n = 3 independent samples). *P < 0.05, **P < 0.01, ***P < 0.001, two-sided Student's t-test. For detailed statistical analysis see Supplementary Table 7. Source data are provided as a Source Data file.

Similarly, 9 nm and 15 nm L- or D-GSH coated Au NPs also exhibit strong inhibition effect against Aβ42 fibrillization, in which the maximum ThT intensity decreases by 36% (L9), 58% (D9), 27% (L15) or 51% (D15) (Supplementary Fig. 7a). Evidently, D9 and D15 show a higher inhibition effect than L9 and L15, respectively. In addition, CD characterization and further analysis of protein secondary structure demonstrate that D9 and D15 are more efficient in preventing the structural transition of Aβ42 from native random coil to β-sheet conformation (Supplementary Fig. 7b,c). AFM and TEM imaging directly shows that the length of the formed Aβ42 fibrils is shorter in the presence of D9 and D15 (Supplementary Fig. 7d,e). Altogether, it could be concluded that the D9 and D15 have better performance on preventing Aβ42 aggregation in comparison with L9 and L15, respectively.

Supplementary Table 4. Values of Lag time, the time required to reach half of the maximum fluorescence intensity $t_{1/2}$ and apparent aggregation constant k obtained for the experiment shown in Fig. 1a and Supplementary Fig. 7a.

Sample	Lag time (h)	$t_{1/2}$ (h)	k (h^{-1})
Aβ42	11.9	18.5 ± 0.3	0.30 ± 0.01
Aβ42 + L3.3	20.9	37.4 ± 5.4	0.12 ± 0.02
Aβ42 + D3.3	26.3	47.4 ± 5.2	0.09 ± 0.01
Aβ42 + C3.5	12.0	31.7 ± 7.6	0.10 ± 0.03
Aβ42 + D9	23.4	42.3 ± 6.1	0.18 ± 0.05
Aβ42 + L9	16.1	27.1 ± 1.7	0.10 ± 0.02
Aβ42 + D15	20.3	38.4 ± 4.2	0.11 ± 0.02
Aβ42 + L15	15.6	21.9 ± 0.8	0.32 ± 0.08

The lag time, $t_{1/2}$ and k can be obtained by fitting the ThT kinetic data with sigmoidal curves (Supplementary Equation 9 and Supplementary Equation 10)[8]:

$$y = y_0 + \frac{y_{\max} - y_0}{1 + e^{-(t - t_{1/2})k}} \tag{9}$$

$$\text{lag time} = t_{1/2} - \frac{2}{k} \tag{10}$$

where y is the fluorescence intensity at time t, y_0 and $y_{\max}$ are the initial and maximum fluorescence intensity, respectively, $t_{1/2}$ is the time required to reach half of the maximum fluorescence intensity (halfway from nuclei to fibrils), and k is the apparent first-order aggregation constant. The lag time is defined as the time, at which the tangent at the point of the maximum fibrillization rate intersects the abscissa. As can be seen in Supplementary Table 4, the chiral Au NPs exhibit inhibition effect on Aβ42 aggregation by increasing the lag time and reducing the rate of Aβ42 aggregation.

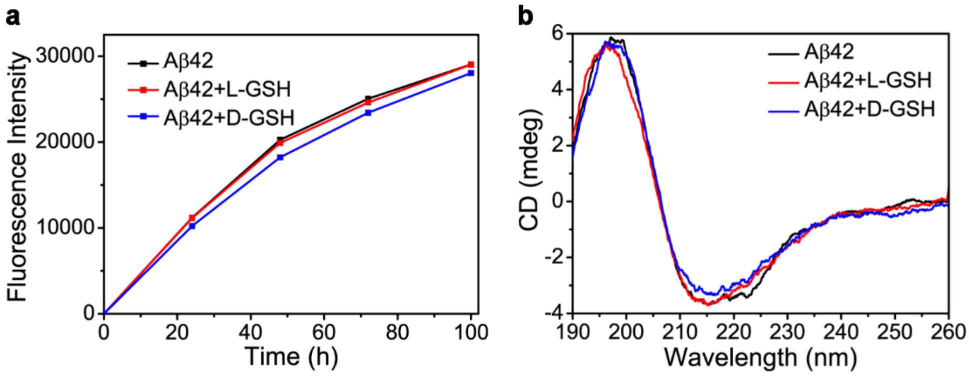

Supplementary Figure 8. Effect of L-GSH and D-GSH molecules on Aβ42 fibrillization in vitro. **a**, Fibrillization kinetics of Aβ42 as monitored by ThT fluorescence assay in the absence (black line) and presence of L-GSH (red line) or D-GSH molecules (blue line) (1 μM). **b**, CD spectra of Aβ42 in the absence (black line) and presence of L-GSH (red line) and D-GSH molecules (blue line) (1 μM) after incubation for 48 h. Source data are provided as a Source Data file.

Obviously, pure L- or D-GSH molecules show negligible inhibition effect on Aβ42 fibrillization due to their weaker interaction with Aβ42 in solution[9].

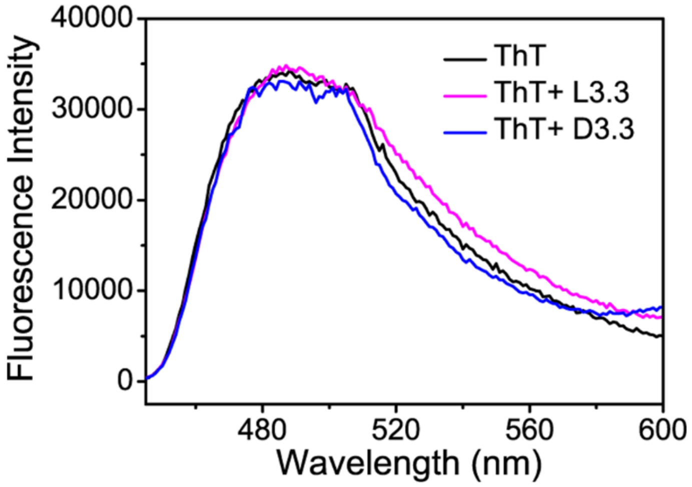

Supplementary Figure 9. Effect of L3.3 and D3.3 on the fluorescence of ThT. Fluorescence spectra of ThT aqueous solution (2 μM) in the absence (black line) and presence of L3.3 (110 nM, magenta line) or D3.3 (110 nM, blue line). The excitation wavelength was 430 nm. Source data are provided as a Source Data file.

As shown in Supplementary Fig. 9, the added L3.3 or D3.3 does not cause an obvious decrease in the fluorescence of ThT solution. Thus, we conclude that the quenching raised by gold nanoparticles under the experimental conditions can be ignored.

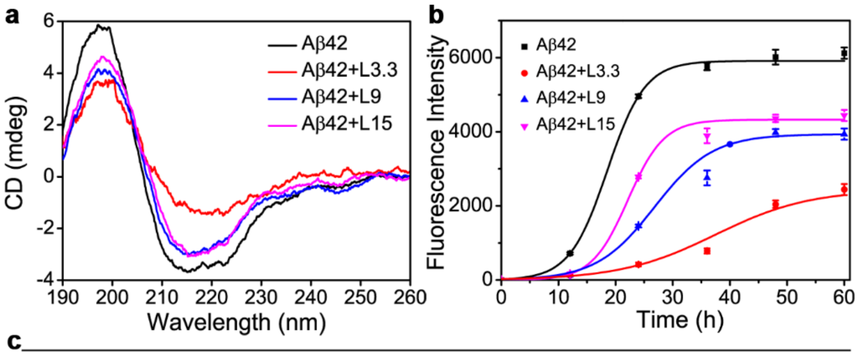

c

Sample	Lag time (h)	$t_{1/2}$ (h)	k (h^{-1})
Aβ42	11.9	18.5 ± 0.3	0.30 ± 0.01
Aβ42 + L3.3	20.9	37.4 ± 5.4	0.12 ± 0.02
Aβ42 + L9	16.1	27.1 ± 1.7	0.10 ± 0.02
Aβ42 + L15	15.6	21.9 ± 0.8	0.32 ± 0.08

Supplementary Figure 10. Size effect of L-GSH coated Au NPs on Aβ42 fibrillization in vitro. **a**,**b**, CD spectra (**a**) and ThT fluorescence assay (**b**) of Aβ42 in absence and presence of L3.3, L9 or L15. Mean ± s.d. values of ThT assay are 14.3 ± 7.8, 718.7 ± 30.7, 4960.0 ± 50.1, 5764.3 ± 100.3, 6016.7 ± 199.9, 6125.0 ± 150.0 at 0, 12, 24, 36, 48, 60 h for Aβ42. Mean ± s.d. values are 6.3 ± 2.1, 125.0 ± 20.1, 421.0 ± 50.5, 785.3 ± 69.6, 2046.0 ± 100.5, 2442.3 ± 150.3 at 0, 12, 24, 36, 48, 60 h for Aβ42+L3.3. Mean ± s.d. values are 8.3 ± 4.0, 145.7 ± 47.0, 1460.3 ± 40.0, 2756.3 ± 199.8, 3974.0 ± 99.8, 3938.3 ± 151.2 at 0, 12, 24, 36, 48, 60 h for Aβ42+L9. Mean ± s.d. values are 10.0 ± 4.6, 125.3 ± 30.2, 527.3 ± 40.1, 958.3 ± 49.7, 2403.3 ± 99.2, 2585.7 ± 150.0 at 0, 12, 24, 36, 48, 60 h for Aβ42+L15 (n = 3 independent samples). **c**, A summary result of lag time, the time required to reach half of the maximum fluorescence intensity $t_{1/2}$ and apparent aggregation constant k obtained by fitting ThT data shown in (**b**) with sigmoidal curves. Source data are provided as a Source Data file.

The L3.3 displays the best efficiency of inhibiting the structural transition of Aβ42 from native random coil to β-sheet conformation (Supplementary Fig. 10a). The lag time, $t_{1/2}$ and k can be obtained by fitting the ThT kinetic data with sigmoidal curves (Supplementary Equation 9 and Supplementary Equation 10). Compared with L9 and L15, L3.3 can effectively inhibit Aβ42 growth process by increasing the lag time and reducing the rate of Aβ42 nucleation (Supplementary Fig. 10b,c).

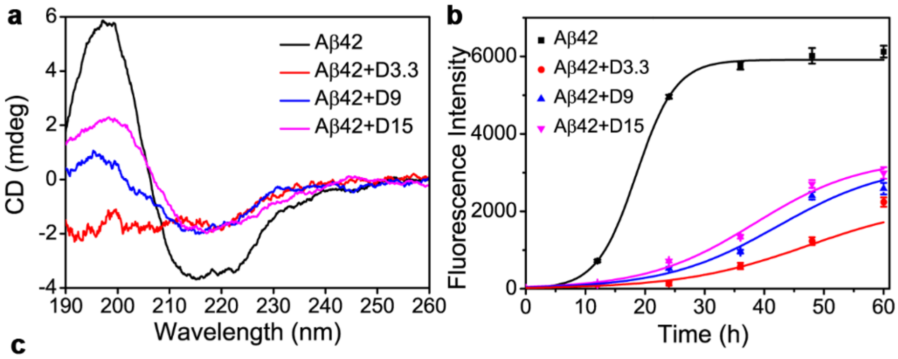

c

Sample	Lag time (h)	$t_{1/2}$ (h)	k (h^{-1})
Aβ42	11.9	18.5 ± 0.3	0.30 ± 0.01
Aβ42 + D3.3	26.3	47.4 ± 5.2	0.09 ± 0.01
Aβ42 + D9	23.4	42.3 ± 6.1	0.18 ± 0.05
Aβ42 + D15	20.3	38.4 ± 4.2	0.11 ± 0.02

Supplementary Figure 11. Size effect of D-GSH coated Au NPs on Aβ42 fibrillization in vitro. **a**,**b**, CD spectra (**a**) and ThT fluorescence assay (**b**) of Aβ42 in absence and presence of D3.3, D9 or D15. Mean ± s.d. values of ThT assay are 14.3 ± 7.8, 718.7 ± 30.7, 4960.0 ± 50.1, 5764.3 ± 100.3, 6016.7 ± 199.9, 6125.0 ± 150.0 at 0, 12, 24, 36, 48, 60 h for Aβ42. Mean ± s.d. values are 8.7 ± 4.0, 105 ± 30, 134 ± 50.2, 587.3 ±79.8, 1233.3 ± 99.6, 2239 ±120.2 at 0, 12, 24, 36, 48, 60 h for Aβ42+D3.3. Mean ± s.d. values are 20.3 ± 18.9, 159.3 ± 49.2, 2781.3 ± 40.5, 3894.0 ± 199.5, 4362.0 ± 99.7, 4441.7 ± 149.6 at 0, 12, 24, 36, 48, 60 h for Aβ42+D9. Mean ± s.d. values are 15.0 ± 11.5, 128.0 ± 30.1, 715.0 ± 39.8, 1346.0 ± 50.3, 2705.0 ± 100.1, 2991.0 ± 150.0 at 0, 12, 24, 36, 48, 60 h for Aβ42+D15 (n = 3 independent samples). **c**, Summary results of lag time, the time required to reach half of the maximum fluorescence intensity $t_{1/2}$ and apparent aggregation constant k obtained by fitting ThT data shown in (**b**) with sigmoidal curves. Source data are provided as a Source Data file.

Same with the size effect of L-GSH-AuNPs on Aβ42 fibrillization in vitro, the D3.3 shows the best performance on preventing the structural transition of Aβ42 from native random coil to β-sheet conformation (Supplementary Fig. 11a). The ThT fluorescence assay and further analysis of the fibrillization kinetics by fitting ThT data with sigmoidal curves clearly show that D3.3 can effectively increase the lag time and reduce the rate of Aβ42 nucleation (Supplementary Fig. 11b,c).

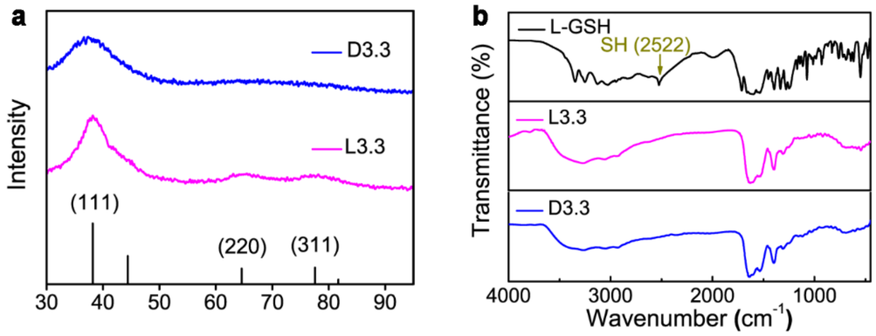

Supplementary Figure 12. Structure and surface characterization of L3.3 and D3.3. a, XRD patterns of as-synthesized L3.3 and D3.3. The standard pattern of Au (PDF#65-2870) is shown by solid bars with Miller indices. **b**, FTIR spectra of L-GSH molecule, L3.3, and D3.3. Deep yellow arrow represents the S-H stretch of L-GSH. Source data are provided as a Source Data file.

The XRD survey shows that the main exposed faces of the synthesized L3.3 and D3.3 are (111) crystal planes (Supplementary Fig. 12a). FTIR spectroscopy demonstrates that the chiral GSH molecules are coated on Au NPs surface via Au-S bonds (Supplementary Fig. 12b).

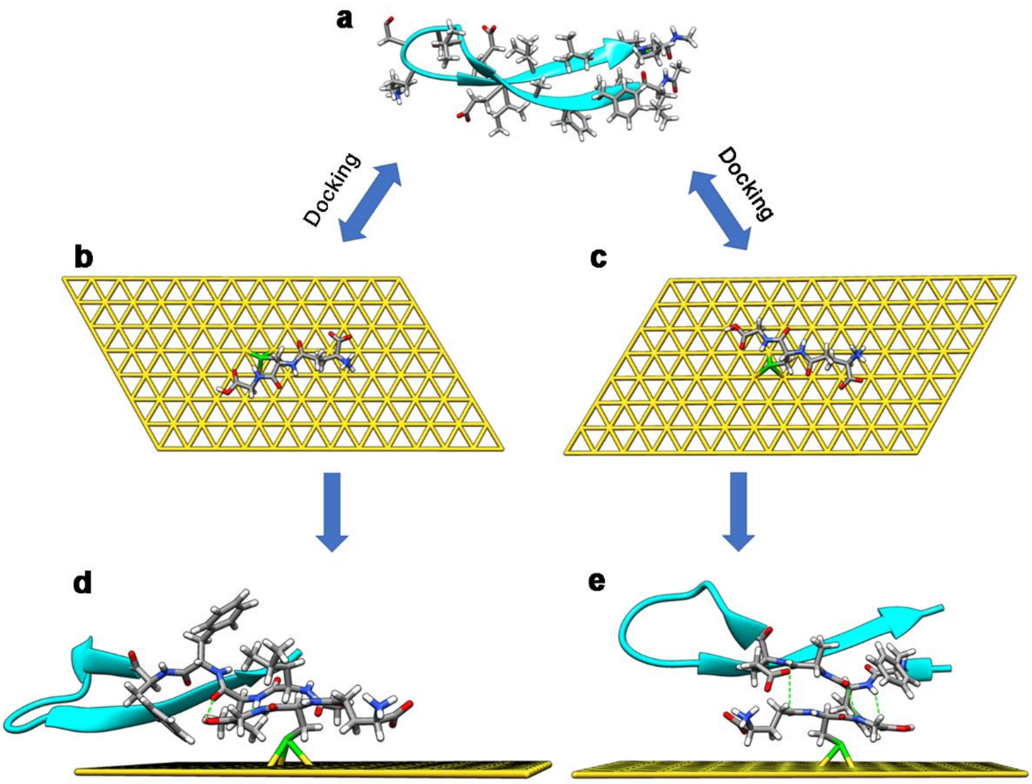

Supplementary Figure 13. DFT computation and molecular docking. **a**, Molecular structure of Aβ17-36 peptide. **b**,**c**, Optimized models of one L-GSH (**b**) or D-GSH molecule (**c**) decorated Au (111) layer through DFT calculation. **d**,**e**, The most stable structures of Aβ17-36 with L- (**d**) or D-GSH stabilized Au (111) surface (**e**) obtained from molecular docking simulation (atom color: C, grey; H, white; N, blue; O, red; S, green; Au, yellow). Green dotted lines represent hydrogen bonding.

To simplify the calculation, the Aβ17-36 crystal structure is used to mimic Aβ42 because they have similar "beta-turn-beta" structure and assembly behavior[10] (Supplementary Fig. 13a). A model of Au (111) layer decorated with one L- or D-GSH molecule via Au-S bond represents L/D3.3 based on their XRD patterns and FTIR spectra (Supplementary Fig. 12). The L- or D-GSH stabilized Au (111) surface is first optimized by DFT calculation (Supplementary Fig. 13b,c), and then docked with Aβ17-36 peptide for 100 times to figure out the most stable binding conformation (Supplementary Fig. 13d,e).

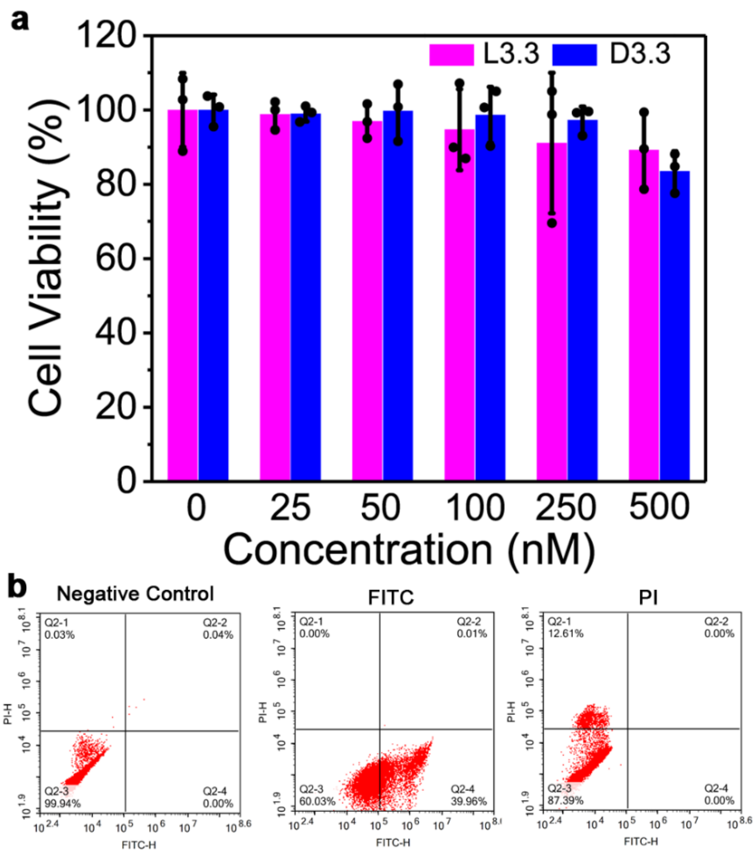

Supplementary Figure 14. Inhibitory effect of L3.3 and D3.3 on Aβ42 aggregation in vitro. a, Relative viability of SH-SY5Y cells treated with L3.3 and D3.3 at various concentrations after 48 h. Cell viability is measured using the CCK-8 assay. Mean ± s.d. values are 100.0% ± 9.9%, 98.9% ± 3.8%, 97.0% ± 4.6%, 94.7% ± 10.9%, 91.1% ±18.9%, 89.2% ± 10.3% for 0, 25, 50, 100, 250, 500 nM of L3.3. Mean ± s.d. values are 100% ± 4.1%, 98.9% ± 2.1%, 99.7% ± 7.7%, 98.6% ± 7.5%, 97.2% ± 3.6%, 83.5% ± 5.3% for 0, 25, 50, 100, 250, 500 nM of D3.3. (*n* = 3 independent samples). **b**, Gating data for flow cytometry. Source data are provided as a Source Data file.

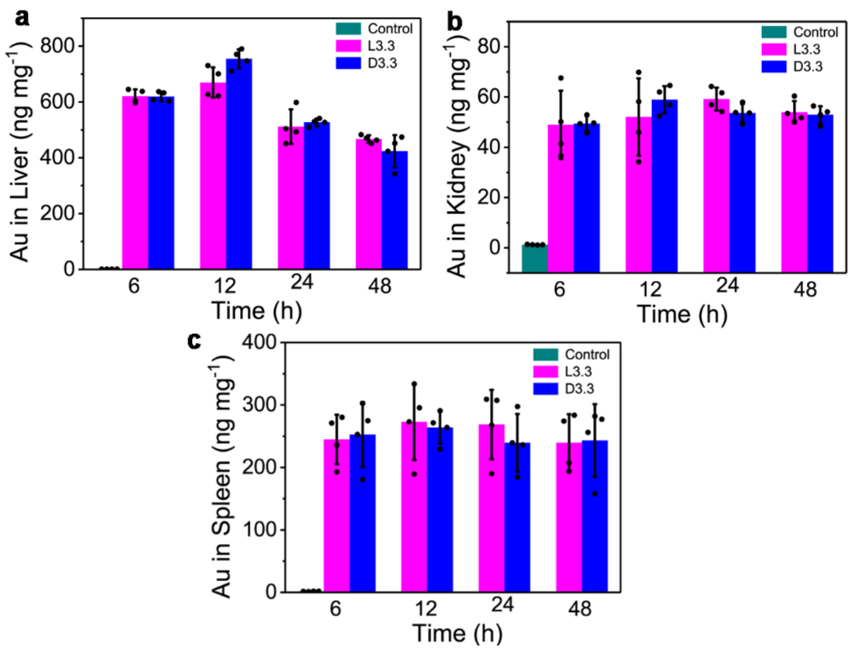

Supplementary Figure 15. Biodistribution analysis of L3.3 and D3.3 in vivo. a, Biodistribution of L3.3 and D3.3 in the liver at 6 h, 12 h, 24 h, and 48 h post-injection. Mean ± s.d. value is 1.513 ± 0.124 for control group. Mean ± s.d. values are 620.598 ± 24.006, 669.590 ± 54.029, 511.585 ± 61.720, 467.115 ± 13.385 at 6, 12, 24, 48 h for L3.3. Mean ± s.d. values are 619.706 ± 17.210, 754.467 ± 33.961, 526.991 ± 13.323, 423.252 ± 57.584 at 6, 12, 24, 48 h for D3.3. **b,** Biodistribution of L3.3 and D3.3 in the kidney at 6 h, 12 h, 24 h, and 48 h post-injection. Mean ± s.d. value is 1.248 ± 0.117 for control group. Mean ± s.d. values are 48.938 ± 13.618, 52.056 ± 15.373, 59.222 ± 4.527, 53.951 ± 4.462 at 6, 12, 24, 48 h for L3.3. Mean ± s.d. values are 49.409 ± 2.946, 58.959 ± 5.381, 53.644 ± 3.552, 52.944 ± 3.394 at 6, 12, 24, 48 h for D3.3. **c,** Biodistribution of L3.3 and D3.3 in the spleen at 6 h, 12 h, 24 h, and 48 h post-injection. Mean ± s.d. value is 1.816 ± 0.278 for control group. Mean ± s.d. values are 244.887 ± 39.555, 272.816 ± 60.858, 268.508 ± 55.643, 239.712 ± 45.460 at 6, 12, 24, 48 h for L3.3. Mean ± s.d. values are 252.582 ± 52.027, 263.821 ± 25.557, 239.466 ± 46.064, 243.197 ± 57.922 at 6, 12, 24, 48 h for D3.3. (*n* = 4 mice per group). Source data are provided as a Source Data file.

The Au content in the liver and spleen is much higher than that in the kidney, indicating that both L3.3 and D3.3 are majorly cleared by the renal system[11].

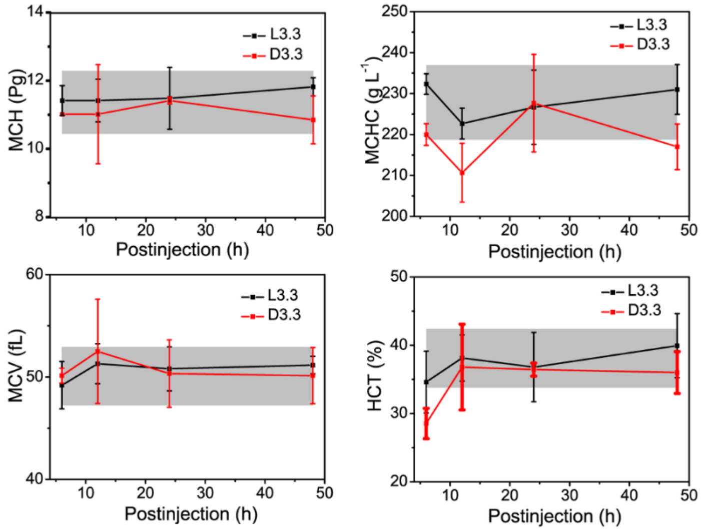

Supplementary Figure 16. Main hematological parameters, including mean corpuscular hemoglobin content (MCH), mean corpuscular hemoglobin concentration (MCHC), mean cell volume (MCV) and hematocrit (HCT) of KM mice after treatment with L3.3 or D3.3 for 6 h, 12 h, 24 h, and 48 h. The shadow regions show the normal range of control groups. Error bars indicate the s.d. (*n* = 3 mice per group). Mean ± s.d. value of MCH is 11.4 ± 0.9 for control group. Mean ± s.d. values of MCH are 11.4 ± 0.4, 11.4 ± 0.6, 11.5 ± 0.9, 11.8 ± 0.3 at 6, 12, 24, 48 h for L3.3. Mean ± s.d. values of MCH are 11.0 ± 0.0, 11.0 ± 1.5, 11.4 ± 0.1, 10.8 ± 0.7 at 6, 12, 24, 48 h for D3.3. Mean ± s.d. value of MCHC is 227.9 ± 9.2 for control group. Mean ± s.d. values of MCHC are 232.3 ± 2.5, 222.7 ± 3.8, 226.7 ± 9.1, 231.0 ± 6.1 at 6, 12, 24, 48 h for L3.3. Mean ± s.d. values of MCHC are 220.0 ± 2.6, 210.7 ± 7.2, 227.7 ± 11.9, 217 ± 5.6 at 6, 12, 24, 48 h for D3.3. Mean ± s.d. value of MCV is 50.1 ± 2.9 for control group. Mean ± s.d. values of MCV are 49.2 ± 2.3, 51.3 ± 2.0, 50.8 ± 2.2, 51.2 ± 0.9 at 6, 12, 24, 48 h for L3.3. Mean ± s.d. values of MCV are 50.1 ± 0.7, 52.5 ± 5.1, 50.3 ± 3.3, 50.1 ± 2.7 at 6, 12, 24, 48 h for D3.3. Mean ± s.d. value of HCT is 38.1 ± 4.3 for control group. Mean ± s.d. values of HCT are 34.6 ± 4.5, 38.1 ± 3.4, 36.8 ± 5.1, 39.9 ± 4.7 at 6, 12, 24, 48 h for L3.3. Mean ± s.d. values of HCT are 28.5 ± 2.2, 36.8 ± 6.3, 36.4 ± 1.0, 36.0 ± 3.1 at 6, 12, 24, 48 h for D3.3. Source data are provided as a Source Data file.

The hematological parameters first suffer slight fluctuation at the early stage of injection (6 h and 12 h), then recover to the normal range (from 24 h to 48 h).

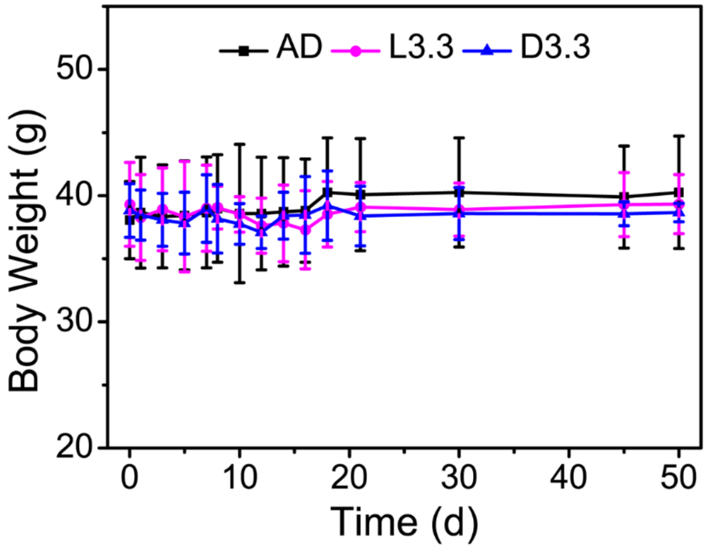

Supplementary Figure 17. Average body weight of AD mice after different treatments. Error bars indicate the s.d. (*n* = 6 mice per group). Mean ± s.d. values are 38.06 ± 3.06, 38.66 ± 3.32, 38.36 ± 3.54, 38.43 ± 3.63, 38.67 ± 3.94, 38.98 ± 4.39, 38.58 ± 4.45, 38.58 ± 4.17, 38.71 ± 3.99, 38.81 ± 3.59, 40.25 ± 3.96, 40.07 ± 3.26, 40.44 ± 3.57, 39.89 ± 4.16, 40.26 ± 4.33 at 0, 1, 3, 5, 7, 8, 10, 12, 14, 16, 18, 21, 30, 45, 50 day for AD mice. Mean ± s.d. values are 39.31 ± 0.86, 38.27 ± 0.74, 38.91 ± 0.82, 38.33 ± 0.76, 39.01 ± 1.00, 39.05 ± 1.51, 38.51 ± 1.41, 37.62 ± 2.18, 37.81 ± 3.04, 37.29 ± 3.09, 38.52 ± 2.59, 39.09 ± 1.95, 38.89 ± 1.69, 39.29 ± 1.50, 39.33 ± 2.24 at 0, 1, 3, 5, 7, 8, 10, 12, 14, 16, 18, 21, 30, 45, 50 day for AD mice treated with L3.3. Mean ± s.d. values are 36.82 ± 1.67, 36.46 ± 1.62, 36.08 ± 1.76, 35.83 ± 1.99, 36.98 ± 2.08, 36.18 ± 2.13, 35.76 ± 1.38, 35.09 ± 1.15, 36.41 ± 1.51, 36.47 ± 2.36, 37.21 ± 2.18, 36.38 ± 1.95, 36.58 ± 1.36, 36.56 ± 0.86, 36.66 ± 0.56 at 0, 1, 3, 5, 7, 8, 10, 12, 14, 16, 18, 21, 30, 45, 50 day for AD mice treated with D3.3. Source data are provided as a Source Data file.

During the therapeutic period, the body weight of AD mice is not significantly affected by L3.3 or D3.3 treatment.

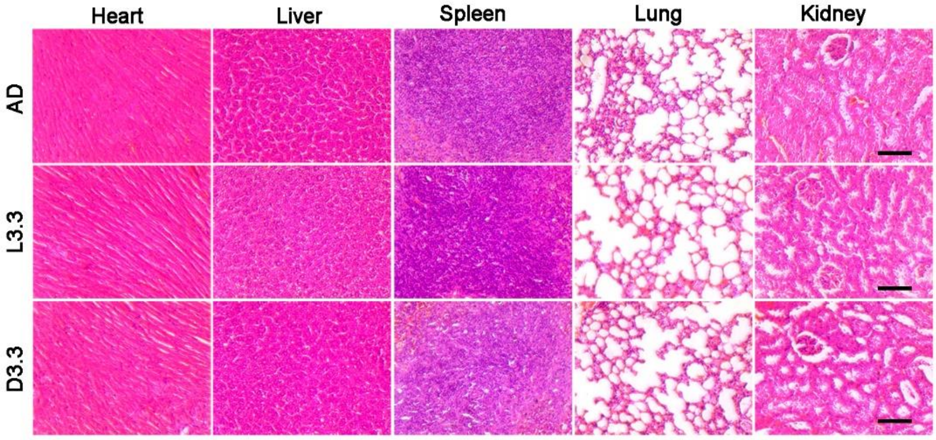

Supplementary Figure 18. Long-term in vivo toxicity of L3.3 and D3.3. Pathological H&E stained images of tissue sections from major organs including heart, liver, spleen, lung, and kidney of AD mice treated with PBS, L3.3 or D3.3 at 45 day post-injection (n = 2 mice per group). Scale bar, 100 μm.

No significant difference is detected in pathological signs between the treated groups and the control groups.

Supplementary Table 5. Comparison of MWM performances of AD mice treated with various NPs.

NPs	Injection method	Target quadrant occupancy				Supplementary References
		WT	AD	NPs	$\frac{NPs}{WT}$	
CeNC/IONC /MSN-T807	Brain stereotaxic injections	30%	14%	25%	83.3%	[12]
CS-$K_{0.5}$-$B_{0.5}$	Intravenous injection with cyclosporine	67%	28%	51%	76.1%	[13]
D3.3	Intravenous injection	39%	24%	37%	94.9%	This work

The reported MWM performances of AD mice treated with various NPs are summarized in Supplementary Table 5. The chiral D3.3 is advantageous not only in the injection method but also in the final performance. In detail, the operation of stereotactic brain injection (Supplementary Reference 12) is complicated and the mortality rate is high. In addition, cyclosporine was used to increase the permeability of BBB through influencing the P-glycoprotein function in Supplementary Reference 13, which might cause serious side effects because many types of substances could also cross the BBB. The performance of mice in the MWM test is evaluated by the target quadrant occupancy in the probe trial. It needs to be noticed that the direct comparison of the target quadrant occupancy is inappropriate because the measured values of WT or AD groups are different in each work. Nevertheless, the target quadrant occupancy of D3.3-treated AD mice is closest to that of the WT group among all the reported NPs (up to 94.9%), indicating the best rescue effect of D3.3.

Supplementary Table 6. Mean ± s.d. values for statistical analyses.

Figure 1a

Time	Aβ42	Aβ42+L3.3	Aβ42+D3.3	Aβ42+C3.5
0 h	14.3 ± 7.8	6.3 ± 2.1	8.7 ± 4.0	5.0 ± 1.7
12 h	718.7 ± 30.7	125.0 ± 20.1	105.0 ± 30.0	627.0 ± 50.0
24 h	4960 ±50.1	421.0 ± 50.5	134.0 ± 50.2	3006.7 ± 100.1
36 h	5764.3 ± 100.3	785.3 ± 69.6	587.3 ±79.8	3450.0 ± 110.0
48 h	6016.7 ± 199.9	2046.0 ± 100.5	1233.3 ± 99.6	4500.0 ± 119.7
60 h	6125 ± 150.0	2442.3 ± 150.3	2239.0 ± 120.2	5432.3 ± 150.1

Figure 1c

Sample	Helix	Beta	Turn	Random
Aβ42	17.6 ± 3.3%	48.3 ± 3.0%	17.5 ± 1.1%	17.6 ± 1.4%
Aβ42+L3.3	3.6 ± 1.6%%	43.2 ± 0.4%	22.9 ± 1.8%	30.0 ± 0.3%
Aβ42+D3.3	7.8 ± 0.2%	29.3 ± 2.1%	23.2 ±0.5%	38.8 ± 1.7%
Aβ42+C3.5	16.5 ± 1.4%	45.2 ± 3.4%	18.3 ± 2.0%	20.3 ± 0.5%

Figure 3a and Figure 3c

Sample	Figure 3a	Figure 3c
Control	100.0 ± 9.8	6.3 ± 2.1
Aβ42	42.2 ± 4.2	42.9 ± 1.0
Aβ42+L3.3	72.3 ± 8.7	36.2 ± 1.4
Aβ42+D3.3	82.7 ± 2.8	32.1 ± 2.4

Figure 4a and Figure 4b

	Time	Control	L3.3	D3.3
Figure 4a	6 h	0.258 ± 0.062	2.282 ± 0.370	2.931 ± 0.151
	12 h		2.583 ± 0.549	2.898 ± 0.083
	24 h		2.343 ± 0.244	2.892 ± 0.822
	48 h		2.181 ± 0.067	2.353 ± 0.283
Figure 4b	6 h	0.457 ± 0.260	19.428 ± 1.111	21.005 ± 1.127
	12 h		14.789 ± 2.341	15.946 ± 1.376
	24 h		7.020 ± 0.391	6.953 ± 0.877
	48 h		2.510 ± 0.113	2.538 ± 0.561

Figure 4d

	Time	Control	L3.3	D3.3
RBC	6 h	7.62 ± 0.72	5.68 ± 0.47	7.03 ± 0.67
	12 h		7.01 ± 0.80	7.44 ± 0.40
	24 h		7.26 ± 0.34	7.23 ± 0.69
	48 h		7.19 ± 0.30	7.82 ± 0.94
HGB	6 h	87.2 ± 11.7	62.7 ± 5.1	80.6 ± 10.0
	12 h		78.3 ± 18.0	85.3 ± 8.6

	24 h		83.0 ± 3.0	84.0 ± 14.8
	48 h		78.3 ± 6.9	92.4 ± 9.5
PLT	6 h	699 ± 250	328 ± 11	395 ± 141
	12 h		676 ± 324	522 ± 335
	24 h		748 ± 209	850 ± 218
	48 h		750 ± 160	796 ± 249
WBC	6 h	3.1 ± 1.3	1.8 ± 0.5	2.6 ± 1.1
	12 h		1.6 ± 0.8	2.2 ± 1.0
	24 h		2.5 ± 1.2	2.2 ± 1.2
	48 h		2.7 ± 0.8	2.0 ± 1.2

Figure 5

		WT	AD	L3.3	D3.3
Figure 5a	Training day-1	108.28 ± 14.19	113.94 ± 10.49	108.12 ± 2.63	100.05 ± 2.78
	Training day-2	70.44 ± 12.95	112.96 ± 6.42	97.98 ± 17.07	90.94 ± 9.98
	Training day-3	69.78 ± 4.86	114.56 ± 9.43	98.38 ± 11.54	72.76 ± 22.91
	Training day-4	65.61 ± 23.76	110.66 ± 8.18	97.30 ± 6.61	65.02 ± 15.00
	Training day-5	31.22 ± 16.34	109.19 ± 5.93	96.12 ± 12.29	65.79 ± 9.68
Figure 5b	Occupancy (%)	39.65 ± 13.03	24.19 ± 3.87	31.05 ± 5.02	36.53 ± 7.55
Figure 5c	Platform crossings	6.17 ± 2.28	0.17 ± 0.41	2.33 ± 1.86	5.33 ± 2.88
Figure 5f	Soluble Aβ42 (ng mg^{-1})	2.25 ± 0.41	10.98 ± 1.28	8.89 ± 1.05	5.87 ± 1.16
Figure 5g	Insoluble Aβ42 (ng mg^{-1})	12.48 ± 1.66	53.42 ± 2.54	37.37 ± 7.61	31.84 ± 11.03
Figure 5h	Soluble Aβ40 (ng mg^{-1})	13.00 ± 5.29	139.01 ± 19.06	79.50 ± 24.61	45.90 ± 16.31
Figure 5i	Insoluble Aβ40 (ng mg^{-1})	32.80 ± 4.81	350.91 ± 54.22	235.42 ± 83.88	81.09 ± 17.91

Supplementary Table 7. *P* values of statistical analyses.

Figure no.	Sample number of group one	Sample name of group two	*P* value	Label
Fig. 1a	60-Aβ42	60-Aβ42+L3.3	<0.001	***
Fig. 1a	60-Aβ42	60-Aβ42+D3.3	<0.001	***
Fig. 1a	60-Aβ42+L3.3	60-Aβ42+D3.3	0.497	*
Fig. 1c	Helix-Aβ42	Helix-Aβ42+L3.3	0.003	**
Fig. 1c	Helix-Aβ42	Helix-Aβ42+D3.3	0.035	*
Fig. 1c	Helix-Aβ42+L3.3	Helix-Aβ42+D3.3	0.010	**
Fig. 1c	Beta- Aβ42	Beta-Aβ42+D3.3	0.001	***
Fig. 1c	Beta-Aβ42+L3.3	Beta-Aβ42+D3.3	<0.001	***
Fig. 1c	Turn-Aβ42	Turn-Aβ42+L3.3	0.012	*
Fig. 1c	Turn-Aβ42	Turn-Aβ42+D3.3	0.001	**
Fig. 1c	Random-Aβ42	Random-Aβ42+L3.3	0.003	**
Fig. 1c	Random-Aβ42	Random-Aβ42+D3.3	<0.001	***
Fig. 1c	Random-Aβ42+L3.3	Random-Aβ42+D3.3	0.001	**
Fig. 2c	ΔG-L3.3	ΔG-D3.3	0.045	*
Fig. 3a	Aβ42	Aβ42+L3.3	0.002	**
Fig. 3a	Aβ42	Aβ42+D3.3	<0.001	***
Fig. 3a	Aβ42+L3.3	Aβ42+D3.3	0.263	NS
Fig. 3c	Aβ42	Aβ42+L3.3	0.009	**
Fig. 3c	Aβ42	Aβ42+D3.3	0.001	**
Fig. 3c	Aβ42+L3.3	Aβ42+D3.3	0.073	NS
Fig. 4a	6-L3.3	6-D3.3	0.018	*
Fig. 4a	12-L3.3	12-D3.3	0.300	NS
Fig. 4a	24-L3.3	24-D3.3	0.247	NS
Fig. 4a	48-L3.3	48-D3.3	0.283	NS
Fig. 4b	6-L3.3	6-D3.3	0.093	NS
Fig. 4b	12-L3.3	12-D3.3	0.430	NS

Fig. 4b	24-L3.3	24-D3.3	0.894	NS
Fig. 4b	48-L3.3	48-D3.3	0.925	NS
Fig. 5a	5-AD	5-L3.3	1.000	NS
Fig. 5a	5-AD	5-D3.3	0.011	*
Fig. 5a	5-AD	5-WT	<0.001	***
Fig. 5a	5-L3.3	5-D3.3	0.012	*
Fig. 5b	WT	AD	0.001	**
Fig. 5b	AD	L3.3	0.442	NS
Fig. 5b	L3.3	D3.3	0.135	NS
Fig. 5b	AD	D3.3	0.003	**
Fig. 5c	WT	AD	<0.001	***
Fig. 5c	AD	L3.3	0.061	NS
Fig. 5c	L3.3	D3.3	0.035	*
Fig. 5c	AD	D3.3	<0.001	***
Fig. 5f	WT	AD	<0.001	***
Fig. 5f	AD	L3.3	0.384	NS
Fig. 5f	L3.3	D3.3	0.109	NS
Fig. 5f	AD	D3.3	0.014	*
Fig. 5g	WT	AD	0.001	**
Fig. 5g	AD	L3.3	0.121	NS
Fig. 5g	L3.3	D3.3	0.927	NS
Fig. 5g	AD	D3.3	0.029	*
Fig. 5h	WT	AD	<0.001	***
Fig. 5h	AD	L3.3	0.020	*
Fig. 5h	L3.3	D3.3	0.261	NS
Fig. 5h	AD	D3.3	0.001	**
Fig. 5i	WT	AD	<0.001	***
Fig. 5i	AD	L3.3	0.134	NS
Fig. 5i	L3.3	D3.3	0.035	*

Fig. 5i	AD	D3.3	0.001	**
Supplementary Fig. 7a	60-Aβ42	60-Aβ42+L9	<0.001	***
Supplementary Fig. 7a	60-Aβ42	60-Aβ42+D9	<0.001	***
Supplementary Fig. 7a	60-Aβ42	60-Aβ42+L15	<0.001	***
Supplementary Fig. 7a	60-Aβ42	60-Aβ42+D15	<0.001	***
Supplementary Fig. 7a	60-Aβ42+L9	60-Aβ42+D9	0.015	*
Supplementary Fig. 7a	60-Aβ42+L15	60-Aβ42+D15	0.030	*
Supplementary Fig. 7c	Helix-Aβ42	Helix-Aβ42+L9	0.020	*
Supplementary Fig. 7c	Helix-Aβ42	Helix-Aβ42+D9	0.007	*
Supplementary Fig. 7c	Helix-Aβ42	Helix-Aβ42+L15	0.017	*
Supplementary Fig. 7c	Helix-Aβ42	Helix-Aβ42+D15	0.017	*
Supplementary Fig. 7c	Beta-Aβ42	Beta-Aβ42+D9	0.004	**
Supplementary Fig. 7c	Beta-Aβ42+L9	Beta-Aβ42+D9	0.001	**
Supplementary Fig. 7c	Beta-Aβ42+L15	Beta-Aβ42+D15	0.013	*
Supplementary Fig. 7c	Turn-Aβ42	Turn-Aβ42+D9	0.002	**

Supplementary Fig. 7c	Turn-Aβ42	Turn-Aβ42+L15	0.009	**
Supplementary Fig. 7c	Turn-Aβ42	Turn-Aβ42+D15	0.007	**
Supplementary Fig. 7c	Turn-Aβ42+L9	Turn-Aβ42+D9	0.002	**
Supplementary Fig. 7c	Turn-Aβ42+L15	Turn-Aβ42+D15	0.01	*
Supplementary Fig. 7c	Random-Aβ42	Random-Aβ42+L9	<0.001	***
Supplementary Fig. 7c	Random-Aβ42	Random-Aβ42+D9	<0.001	***
Supplementary Fig. 7c	Random-Aβ42	Random-Aβ42+L15	<0.001	***
Supplementary Fig. 7c	Random-Aβ42	Random-Aβ42+D15	<0.001	***
Supplementary Fig. 7c	Random-Aβ42+L9	Random-Aβ42+D9	0.003	**
Supplementary Fig. 7c	Random-Aβ42+L15	Random-Aβ42+D15	0.037	*

Supplementary References

1. Ahmed, M. *et al.* Structural conversion of neurotoxic amyloid-β 1-42 oligomers to fibrils. *Nat. Struct. Mol. Biol.* **17**, 561-567 (2010).

2. Zhang, R. *et al.* Physical gelation of polypeptide-polyelectrolyte-polypeptide (ABA) copolymer in solution. *Macromolecules* **45**, 6201-6209 (2012).

3. Velichko, Y. S., Stupp, S. I. & De La Cruz, M. O. Molecular simulation study of peptide amphiphile self-assembly. *J. Phys. Chem. B* **112**, 2326-2334 (2008).

4. Auer, S., Dobson, C. M. & Vendruscolo, M. Characterization of the nucleation barriers for protein aggregation and amyloid formation. *HFSP J.* **1**, 137-146 (2007).

5. Jain, P. K., Lee, K. S., El-Sayed, I. H. & El-Sayed, M. A. Calculated absorption and scattering properties of gold nanoparticles of different size, shape, and composition: applications in biological imaging and biomedicine. *J. Phys. Chem. B* **110**, 7238-7248 (2006).

6. Sandberg, A. *et al.* Stabilization of neurotoxic Alzheimer amyloid-β oligomers by protein engineering. *Proc. Natl. Acad. Sci.* **107**, 15595-15600 (2010).

7. Laganowsky, A. *et al.* Atomic view of a toxic amyloid small oligomer. *Science* **335**, 1228-1231 (2012).

8. Cabaleiro-Lago, C. *et al.* Inhibition of amyloid beta protein fibrillation by polymeric nanoparticles. *J. Am. Chem. Soc.* **130**, 15437-15443 (2008).

9. Streich, C. *et al.* Characterizing the effect of multivalent conjugates composed of Aβ-specific ligands and metal nanoparticles on neurotoxic fibrillar aggregation. *ACS Nano* **10**, 7582-7597 (2016).

10. Spencer, R. K., Li, H. & Nowick, J. S. X-ray crystallographic structures of trimers and higher-order oligomeric assemblies of a peptide derived from Aβ17-36. *J. Am. Chem. Soc.* **136**, 5595-5598 (2014).

11. Papasani, M. R., Wang, G. & Hill, R. A. Gold nanoparticles: the importance of physiological principles to devise strategies for targeted drug delivery. *Nanomed. Nanotechnol. Biol. Med.* **8**, 804-814 (2012).

12. Chen, L. *et al.* Tau-targeted multifunctional nanocomposite for combinational therapy of Alzheimer's disease. *ACS Nano* **12**, 1321-1338 (2018).

13. Luo, Q. *et al.* A self-destructive nanosweeper that captures and clears amyloid β-peptides. *Nat. Commun.* **9**, 1802 (2018).
