# Supplementary material for: Chiral gold nanoparticles enantioselectively rescue memory deficits in a mouse model of Alzheimer’s disease
Source: Nat Commun. 2020 Sep 22;11:4790. doi: 10.1038/s41467-020-18525-2 (PMC7509831; doi:10.1038/s41467-020-18525-2)
Supplement: Supplementary file 3 — Description of Additional Supplementary Files [file 41467_2020_18525_MOESM3_ESM.pdf]

Description of Additional Supplementary Files

File Name: Supplementary Movie 1
Description: Representative motion path of WT mice on the fifth day of training in MWM test.

File Name: Supplementary Movie 2
Description: Representative motion path of AD mice on the fifth day of training in MWM test.

File Name: Supplementary Movie 3
Description: Representative motion path of L3.3-treated AD mice on the fifth day of training in MWM test.

File Name: Supplementary Movie 4
Description: Representative motion path of D3.3-treated AD mice on the fifth day of training in MWM test.

File Name: Supplementary Movie 5
Description: Representative motion path of WT mice in the probe trial of MWM test.

File Name: Supplementary Movie 6
Description: Representative motion path of AD mice in the probe trial of MWM test.

File Name: Supplementary Movie 7
Description: Representative motion path of L3.3-treated AD mice in the probe trial of MWM test.

File Name: Supplementary Movie 8
Description: Representative motion path of D3.3-treated AD mice in the probe trial of MWM test.
